# Non-parental Care Arrangements, Parenting Stress, and Demand for Infant-Toddler Care in China: Evidence From a National Survey

**DOI:** 10.3389/fpsyg.2021.822104

**Published:** 2022-01-24

**Authors:** Xiumin Hong, Wenting Zhu, Li Luo

**Affiliations:** ^1^Faculty of Education, Institute of Early Childhood Education, Beijing Normal University, Beijing, China; ^2^School of Government, Beijing Normal University, Beijing, China; ^3^College of Preschool Education, Capital Normal University, Beijing, China

**Keywords:** non-parental child care choice, parenting stress, infants and toddlers, nurseries, Chinese families

## Abstract

This study examined the patterns and characteristics of non-parental child care arrangements for Chinese very young children before they enter preschool and the extent to which families’ utilization of non-parental child care influenced parenting stress. A total of 3,842 Chinese parents of infants and toddlers were selected from 10 provinces to participate in this study. The results indicated that (1) Chinese families relied heavily on grandparents to care for their children; (2) a set of family demographics predicted the utilization of non-parental child care arrangements, including parents’ educational level, household income, labor force participation, and maternal age; (3) there existed a clear parental preference for publicly funded, affordable, and high-quality child care services; and (4) families’ use of non-parental child care was generally not linked to parenting stress. These findings shed light on the development of the infant-toddler non-parental child care system in the Chinese sociocultural context.

## Introduction

The first few years of life are one of the most critical time periods, laying the foundation for later development and learning. With the increasing number of mothers of young children participating in the labor force market, non-parental child care has become a common early experience for infants, toddlers, and preschoolers across many countries (UNICEF, 2008). The high utilization of non-parental child care at early ages has changed the daily life experiences of very young children, which introduced more relationships with adults and group activities with peers (Shonkoff and Phillips, 2000). Since the enactment of the two-child policy and the newly promulgated three-child policy in mainland China, along with the social impact of industrialization and urbanization, the traditional mother-dominated child care model cannot be sustained. Among the pressures of child-rearing, Chinese parents are likely to face work-family conflict, and their need for non-parental child care will become even more pressing. However, the country has been facing shortages of infant-toddler child care services, which is one of the most significant barriers to the effective implementation of China’s two-child and three-child policy (Tian et al., 2020; Hong, 2021). The Chinese government has put great efforts into developing child care services for children under age 3 and takes it as an important supporting measure for the three-child policy. In this context, it is necessary to investigate the perceived stress and needs of parents in terms of child care. Although, after the population policy shift, Chinese researchers began to pay attention to child care services, most of the existing studies are either theoretical analyses or introduction of international experience (e.g., Yang, 2018; Guo, 2021). Only few studies so far have examined the actual status of non-parental child care for Chinese infants and toddlers. Thus, this study conducted a comprehensive analysis of non-parental child care arrangements, parenting stress, and demand for child care in the years before preschool, in order to provide reference and inspiration for the improvement of supportive child care services for families with very young children in mainland China.

### Non-parental Child Care for Infants and Toddlers in Mainland China

In mainland China, infant-toddler care services have been marginalized for a long time, and the external family support system has not yet been established. Due to the development of the market-oriented economy in the 1980s, publicly funded nurseries (as part of welfare benefits to support dual-earner couples by their employment units such as schools, factories, and hospitals) have been either shut down or migrated to private centers, and the responsibility of caring for infants and toddlers has returned to the family (Qi and Melhuish, 2017; Hong and Tao, 2019). According to the national population and family dynamic monitoring data of China, only 5.5% of children under age 3 have been enrolled in nurseries (Hong, 2020), which is far lower than the preschool enrollment rate for children aged 3–6 years. With the preschool expansion movement since 2010, the gross enrollment rate of the 3-year preschool education has reached 83.4% by 2019 nationwide (Ministry of Education of the People’s Republic of China, 2020).

In contrast to the scarce supply of child care services in the 0- to 3-year-old market, Chinese families’ demand for infant-toddler child care is increasing (Hong and Tao, 2019). Especially since the two-child policy was introduced in 2016, there exists booming demand for child care services for young children prior to preschool entry. One-third of parents surveyed in 10 cities and 48% of parents surveyed in four provinces reported having such demand (Yang, 2018). Furthermore, a report by the National Health Commission of the People’s Republic of China (PRC) suggests nearly 61% of the working mothers surveyed were reluctant to have a second child because they have no one to care for their children, and the inadequate supply of child care services for infants and toddlers has become a major barrier affecting fertility intent under the two-child policy (National Health Commission of the People’s Republic of China, 2017). To address the supply-demand mismatch and to boost fertility, the central and local governments in mainland China are establishing the system of infant-toddler child care services with policy support and standards. In recent years, extensive efforts have been made by the Chinese government to support the development of various types of nurseries for infants and toddlers, as well as to strengthen the supervision over nurseries and improve the quality of child care services (Hong and Tao, 2019).

In the face of huge care difficulties, many families choose to seek grandparents for taking care of their children. Child care provided by grandparents has long been a staple of family support in mainland China (Chen et al., 2011). Data from a national survey indicated that 72% of urban elderly women participated in caring for their grandchildren (China Research Center on Aging [CRCA], 2012). Compared with preschool-aged children, the prevalence of grandparental care is higher for infants and toddlers (Chen et al., 2011). A study conducted by the Shanghai Population and Family Planning Committee found that 53% of infants and toddlers in 34 Shanghai communities were primarily cared for by their grandparents, even though 70% of these families showed dissatisfaction with this type of care (Nyland et al., 2009). Although the use of center-based care and grandparental care is not mutually exclusive, research suggests that Chinese families’ demand for grandparent-provided care was higher when center-based care was less accessible or more expensive (Du et al., 2019).

### Parental Selection of Non-parental Child Care

Given the increased rate of dual-earner families, there exists a great demand for non-parental care for young children. It is crucial to understand the parental selection of non-parental care and to examine the influencing factors of child care choices (Kensinger Rose and Elicker, 2008). Existing studies largely focus on exploring the parental selection of the type of child care (e.g., center-based care vs. family-based care) and the cost of child care (e.g., Hofferth and Wissoker, 1992; Meyers and Jordan, 2006). The National Household Education Survey of Early Childhood Program Participation (NHES-ECPP) in the United States is a comprehensive and detailed investigation, which not only includes the type of care a child was receiving but also surveys how the children’s parents find and choose the care. Based on the 2005 NHES-ECPP data, Kim and Fram (2009) identified four distinct profiles of parental priorities in choosing child care and found these patterns were associated with family sociodemographic factors, such as socioeconomic status, employment, ethnicity, and child’s age.

Parents’ choice of child care arrangements reflects a dynamic and complex decision process in the context of the resources, needs, and constraints of families (Carlin et al., 2019). A substantial body of studies has been carried out to identify the determining factors of parents’ choice of either a specific type of non-parental care or a particular child care center (e.g., Gamble et al., 2009), which primarily focused on the features of child care setting (Leslie et al., 2000). Johansen et al. (1996) further divided the attributes of child care into (1) intrinsic characteristics affecting the child’s experience, such as curriculum, age-appropriate toys, and group size; and (2) extrinsic characteristics influencing the parents’ experience, such as location, hours of operation, and cost. While numerous studies have shown that intrinsic characteristics were very important to parents in their selection of care (Leslie et al., 2000), extrinsic features of child care were given more weight in some cases. For example, convenience was considered a major factor in the parental selection of child care for their infants and toddlers (Ahn and Shin, 2013). From an ecological perspective, Seo (2003) proposed a theoretical model that relates non-parental care selection to (1) the characteristics of the child, such as the child’s age and status of special needs; (2) the characteristics of the family, such as household income and maternal education; (3) parents’ beliefs about child care; and (4) environmental context variables, such as family’s social network and child care program characteristics.

### Non-parental Child Care and Parenting Stress

The utilization of non-parental child care can relieve parents of their caregiving responsibility for a significant amount of time, which has the potential to reduce parents’ parenting stress. However, some studies suggested that the utilization of non-parental child care in the year prior to school has not been accompanied by a decrease in parenting stress (e.g., Magnuson and Waldfogel, 2005). Based on the data from the Household, Income and Labor Dynamics in Australia survey, Craig and Churchill (2018) found that longer non-parental child care was related to greater levels of parenting stress of dual-earner couples with young children. Non-parental child care might exacerbate parenting stress due to the associated time demands of organizing child care, scheduling pressures, and the high cost of child care (Craig and Powell, 2013; Pilarz and Hill, 2017). Moreover, parents’ worries about their children’s health and safety in non-parental child care might add to parents’ mental stress (Craig and Churchill, 2018).

Instability of non-parental child care, including changes in care arrangements, multiple arrangements, and needing to use backup arrangements, leads to elevated levels of parenting stress (Chaudry, 2004). Specifically, changes in child care arrangements disrupt the routines in a household, which require parents to reconcile work and family demands; multiple arrangements add to the burden of transportation across different caregivers and coordinating work-family schedules; and the use of a “backup” provider is typically due to the unavailability of their regular provider, indicating the unpredictability in child care arrangements (Pilarz and Hill, 2017). Researchers also examined the linkages between the type of non-parental care arrangements and parenting stress, suggesting that informal and family care arrangements might lower the levels of parenting stress (e.g., Craig and Churchill, 2018; Hwang and Jung, 2020).

### Current Study

Given the significance of non-parental child care and considerable emphasis in recent years on the infant-toddler child care services in mainland China, it is important to understand families’ current utilization of non-parental care arrangements for their children and preferred care characteristics. However, the vast majority of studies on non-parental child care have been conducted in Western countries, where the regulatory policies and context of child care vary. Furthermore, very few Chinese studies have focused on young children’s participation in non-parental care before they enter preschool in the new era of China’s three-child policy. The purposes of this study were to (1) investigate Chinese families’ utilization of non-parental care for their children in the years before preschool and their preferred features of nurseries and (2) explore the linkage between the utilization of non-parental care and parenting stress. Specifically, this study focuses on the following three research questions:

Research Question 1: What about the utilization of non-parental care arrangements for children from birth through age 3? What family-related demographic factors were associated with the non-parental care arrangements?

Research Question 2: What were parents’ demands for nurseries? What factors constrain their child care choices?

Research Question 3: Were children’s participation in non-parental care arrangements associated with parenting stress?

## Materials and Methods

### Participants

This study used a cross-sectional, descriptive survey design. Stratified sampling was used to recruit Chinese parents of children from birth through age 3 who had not yet been enrolled in preschools. First, we selected ten provinces in order to represent eastern, central, and western China: Beijing, Shanghai, Fujian, Guangdong, Henan, Shanxi, Guizhou, Xinjiang, Sichuang, and Yunnan. Second, within each participating province, one or two cities were selected, which resulted in 12 cities. Third, given that it is a central government-funded study, local educational authorities were requested to disseminate the questionnaires by using their available online communication channels and resources. A total of 4,200 questionnaires were sent out, and 3,842 were returned, indicating a response rate of 91.5%.

Our study participants were diverse in terms of the geographical region: 34.3% were from the eastern region, 36.0% were from the central region, and 29.7% were from the western region. Among these parents, more than 60% of them reported having a child aged 37–48 months. In these families, there were 2,039 (53.1%) boys and 1,803 (46.9%) girls, and 3,521 (91.6%) of whom were the only children in their family. As shown in Table 1, the majority of fathers (53.2%) and mothers (54.1%) had a bachelor’s or higher degree. Near half of the fathers (49.2%) and mothers (49.3%) were between the ages of 31 and 35. With respect to household income, about one-third of the families reported monthly income as a medium level of 7,500–15,000 RMB (34.1%). The majority of the households were dual income (79.1%).

**TABLE 1:** Participant characteristics (*N* = 3,842).

Demographic characteristic	Codes in SPSS	Frequency	Percentage (%)
**Child’s gender**			
Male	0	2,039	53.1
Female	1	1,803	46.9
**Child’s age**			
Less than 2 years old	0	462	12.0
2–3 years old	1	890	23.2
3–4 years old	2	2,490	64.8
**Singleton**			
Only children	0	3,521	91.6
Not only children	1	321	8.4
**Mother’s educational level**			
Less than high school	0	327	8.5
High school	1	559	14.5
Associate’s degree	2	877	22.8
Bachelor’s degree	3	1,675	43.6
Master’s degree or above	4	404	10.5
**Father’s educational level**			
Less than high school	0	325	8.5
High school	1	633	16.5
Associate’s degree	2	837	21.8
Bachelor’s degree	3	1,588	41.3
Master’s degree or above	4	459	11.9
**Household income (monthly)^a^**			
Low (Less than 7,500 RMB)	0	1,433	37.3
Medium (7,501 RMB to 15,000 RMB)	1	1,312	34.1
High (15,001 RMB or higher)	2	1,097	28.6
**Labor force status**			
One parent in labor force	0	6,67	17.4
Both parents in labor force	1	3,040	79.1
Other	2	135	3.5
**Mother’s age**			
Less than 30 years old	0	1,316	34.3
31–35 years old	1	1,896	49.3
36 years old or older	2	630	16.4
**Father’s age**			
Less than 30 years old	0	854	22.2
31–35 years old	1	1,892	49.2
36 years old or older	2	1,096	28.5

*^a^High, medium, and low household incomes are based on the per capita disposable income nationwide in 2020 (National Bureau of Statistics of China [NBSC], 2021).*

### Measures

#### Demographic Questionnaire

The purpose of the demographic questionnaire was to collect the child and his/her parents’ basic demographic information, including the child’s age and gender, parental educational level, labor force status of parents, age of parents, number of children in the household, and family income.

#### Adapted Version of the ECPP Survey

The ECPP survey has been administered as an important part of the National Household Education Surveys Program since 1991, which is sponsored by the National Center for Education Statistics of the United States Department of Education. The ECPP survey is designed as a nationally representative study to investigate young children’s care and education before they enter kindergarten in the United States (Corcoran and Steinley, 2019). The adapted version of the 2016 ECPP survey was used in this study, which included two sections: children’s participation in non-parental care arrangements and parents’ demand for nurseries. In the first section, we retained the ECPP questions about the type of care: (1) “Is your child regularly receiving care from a relative other than the child’s parent or guardian”; (2) “Is your child regularly receiving care from someone who is not a relative of your family?”; and (3) “Is your child regularly attending a nursery?” Each question was then followed by a detailed inquiry, such as the care provider and place setting. In the second section, we developed four multiple choice questions about parents’ preferences for the characteristics of nurseries, including the type, location, cost, and distance between home and institution (e.g., “What is your preferred type of nursery?”). In addition, we modified three ECPP questions about finding and choosing center-based care, i.e., “what is the key reason your family wanted a nursery for this child?,” “how much difficulty was it for you to find a nursery for this child?,” and “what was the primary cause for the difficulty in finding a nursery?” Parents are the respondents.

#### Parenting Stress Index – Short Form

In this study, parents were requested to rate their level of stress in the parent-child system using the short form of the Parenting Stress Index (PSI-SF; Abidin, 1995). The PSI-SF consists of 36 items organized under three subscales: (1) Parental Distress subscale, which assesses a parent’s distress as a result of personal factors related to parenting; (2) Parent-Child Dysfunctional Interaction subscale, which measures a parent’s views of their child’s failure to meet expectations; and (3) Difficulty Child subscale, which assesses a parent’s perception of their child’s non-compliance, defiance, difficult temperament, and demanding. Each subscale contains 12 items that are rated on a 5-point Likert-type scale, ranging from 1 (*strongly disagree*) to 5 (*strongly agree*). Thus, the range of subscale scores is 12–60, and the range of total scores is 36–180. The greater level of parenting stress is indicated by a higher score on the PSI-SF. The PSI-SF has been validated to be appropriate for young children’s parents in both the Western (e.g., Reitman et al., 2002; Rivas et al., 2020) and the Chinese contexts (e.g., Tam et al., 1994; Liu and Wang, 2015). In this study, Cronbach’s alpha for the three subscales of the PSI-SF ranged from 0.92 to 0.94.

### Analytic Strategies

All the data were analyzed by using IBM SPSS Statistics version 26.0 software. First, we described the status of non-parental care arrangements participation by providing descriptive information. In our analyses, non-parental child care referred to any care provided by someone who was not the child’s parents or guardians at least once a week (Harrison et al., 2009). Second, binary logistic regression was conducted to test the associations between family-related demographic factors and children’s participation in non-parental care arrangements. Third, we conducted a multivariate analysis of variance (MANOVA) analysis to test differences in parenting stress between parents of children in non-parental care arrangements and parents who did not use non-parental care arrangements. Fourth, a series of chi-square tests of association were performed to examine whether there was a relationship between parental demand for nurseries and their utilization of non-parental care arrangements.

## Results

### Chinese Children’s Participation in Weekly Non-parental Care Arrangements

In this study, approximately 47.0% of Chinese children from birth through age 3 participated in non-parental child care on a regular basis, as reported by their parents. Specifically, 43.4% of children were receiving relative care, 4.5% were receiving non-relative care, and 3.9% were attending a nursery. Across these three types of care, children may be involved in more than one arrangement. About 4.6% of children were reported to weekly participate in multiple types of non-parental care arrangements.

Among children who were in weekly relative care (*n* = 1,667), 51.3% of them were primarily cared for by a paternal grandparent, 38.7% were cared for by a maternal grandparent, and 10.0% were cared for by another relative (e.g., an aunt or uncle). The average age of these relative caregivers was 57.59 (*SD* = 8.45). The most prevalent location for children’s weekly relative care was children’s home (66.0%), followed by both children’s home and relative caregivers’ home (21.3%), and relative caregivers’ home (12.7%).

Regarding weekly non-relative care (*n* = 173), 43.4% of care providers were live-in caregivers. In terms of the locations of non-relative care, 61.8% took place in children’s homes, 15.0% in care providers’ homes, and 23.1% in both homes. About 74.0% of parents already knew this non-relative care provider before sending children to this care arrangement. However, one-third (34.1%) of parents would recommend this non-relative care provider to another parent.

### Demographic Variables Predicting Children’s Participation in Non-parental Care Arrangements

The logistic regression analysis was conducted to examine whether or not the demographic variables would predict children’s participation in weekly non-parental care arrangements. Table 2 displays the demographic variables, regression coefficients, and odds ratios (ORs). The ORs indicate the likelihood of children’s participation in weekly non-parental care arrangements, while the other variables were held constant in the model. Results of the logistic regression showed that children whose mothers obtained a bachelor’s degree (OR = 1.865, *p* = 0.001) or master’s degree (OR = 2.445, *p* < 0.001) had a higher likelihood of being in non-parental care arrangements, compared to our reference group of children whose mothers did not complete high school. Children whose fathers held an associate degree (OR = 1.558, *p* = 0.017) were more likely to receive non-parental care than peers whose fathers did not complete high school.

**TABLE 2:** Logistic estimates for the likelihood of weekly non-parental care arrangements participation.

Variable	Participation in weekly non-parental care arrangements		
	*B*	*SE*	*OR* [95% CI]
Constant	–1.436	0.173	0.238
Child’s gender (female)	0.009	0.069	1.009 [0.881, 1.155]
Singleton (not only children)	–0.202	0.128	0.817 [0.635, 1.051]
Mother’s educational level (high school)	–0.066	0.184	0.937 [0.653, 1.343]
Mother’s educational level (Associate’s degree)	0.192	0.184	1.211 [0.844, 1.738]
Mother’s educational level (Bachelor’s degree)	0.623**	0.189	1.865 [1.287, 2.704]
Mother’s educational level (Master’s degree or above)	0.894***	0.225	2.445 [1.572, 3.802]
Father’s educational level (high school)	0.180	0.180	1.197 [0.842, 1.703]
Father’s educational level (Associate’s degree)	0.443*	0.186	1.558 [1.082, 2.242]
Father’s educational level (Bachelor’s degree)	0.302	0.190	1.352 [0.932, 1.960]
Father’s educational level (Master’s degree or above)	0.262	0.221	1.300 [0.843, 2.005]
Family income (¥7,501 to ¥ 15,000)	–0.131	0.087	0.878 [0.739, 1.042]
Family income (¥15,001 or higher)	0.431***	0.102	1.539 [1.260, 1.880]
Labor force status of parents (both parents in labor force)	0.820***	0.102	2.270 [1.860, 2.771]
Mother’s age (31–35 years old)	−0.223*	0.103	0.800 [0.653,0.980]
Mother’s age (36 years old or older)	−0.392**	0.148	0.676 [0.506,0.903]
Father’s age (31–35 years old)	0.055	0.115	1.057 [0.844, 1.323]
Father’s age (36 years old or older)	0.142	0.143	1.153 [0.872, 1.525]

B, unstandardized coefficient; S.E., standard error; OR, odds ratio. *p < 0.05. **p < 0.01. ***p < 0.001.

As shown in Table 2, the odds of participating in non-parental care arrangements were greater when children were from dual-income households (OR = 2.270, *p* < 0.001) or from families whose monthly income was more than 15,001 RMB (OR = 1.539, *p* < 0.001). Furthermore, children whose mothers were aged between 31 and 35 years (OR = 0.800, *p* = 0.031) or over age 36 (OR = 0.676, *p* = 0.008) were less likely to participate in non-parental care arrangements, when compared to children whose mothers were under age 30. However, fathers’ age, children’s age, and whether the child was the only child in the household did not statistically significantly predict the probability of children’s participation in non-parental care arrangements.

### Chinese Parents’ Demand for Child Care

**FIGURE 1:**
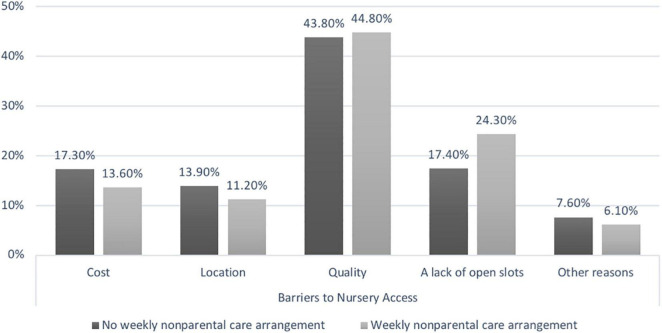
The primary reason for the difficulty finding nurseries as reported by Chinese parents (*N* = 1,945).

In this study, the most common reason^1^ reported by Chinese parents for using center-based care (i.e., the nursery in the Chinese context) was to provide cultural or language learning for children (44.2%), followed by preparing children for school (42.0%), providing care while parents were at work or school (39.7%), and making time for running errands or relaxing (12.5%). However, among parents who reported trying to find a nursery, 68% of parents reported difficulty finding nurseries. For these parents, 44.3% reported that quality of care was the strongest deterrent to finding appropriate nurseries. Other reasons for difficulty finding nurseries included scarcity of available slots for new children (20.7%), cost (15.5%), location (12.6%), and others such as a specific program to meet families’ needs (6.8%). A statistically significant association was observed between the main reason for difficulty finding nurseries and the unitization of non-parental care arrangements [χ^2^ (4,1945) = 19.871, *p* < 0.001]. As shown in Figure 1, lack of available slots was more likely to be reported as the main reason for difficulty finding nurseries by parents of children in non-parental care arrangements than by parents who cared for their children exclusively.

With respect to parental preferences for nursery characteristics, more than 80% of parents reported the need for a publicly funded nursery and expected that the nursery was less than a mile away from their homes. Nearly 60% of parents reported expecting preschool programs to enroll children under age 3, and the preferred cost of child care was below RMB 1,000 per month. While being asked when it was appropriate to send children to nurseries, 51.4% of parents reported 31–36 months old was the expected nursery starting age, followed by 25–30 (26.1%), 19–24 (10.9%), 13–18 (6.7%), 7–12 (3.1%), and under 6 months old (1.9%). There was a statistically significant association between expected nursery starting age and the unitization of non-parental care arrangement [χ^2^(5,3842) = 84.488, *p* < 0.001]. Generally, parents of children in non-parental care arrangements were more likely to expect an earlier nursery starting age than parents who did not use non-parental care arrangements.

### Association Between Non-parental Care Arrangements Participation and Parenting Stress

We conducted a MANOVA analysis to explore the possible effects of children’s participation in non-parental care arrangements on parenting stress. Results reveal that there were no statistically significant differences between parents of children in weekly non-parental care arrangements and their counterparts who did not use weekly non-parental care arrangements for the total PSI-SF scores, the Parental Distress subscale scores, and the Difficulty Child subscale scores. However, the utilization of non-parental care arrangements had a statistically significant effect on the scores of the Parent-Child Dysfunctional Interaction subscale, as shown in Table 3. Parents of children participating in weekly non-parental care arrangements obtained lower scores than parents of children who did not receive weekly non-parental care on the Parent-Child Dysfunctional Interaction subscale [*F*(1,3840) = 5.978, *p* < 0.05, partial η^2^ = 0.002], suggesting that parents who exclusively cared for their children felt greater levels of stress related to the interactions with their children than parents who used weekly non-parental care arrangements.

**TABLE 3:** Comparisons of parenting stress between parents of children in and not in weekly non-parental care arrangements.

	PSI-SF	PD	P-CDI	DC
	*M* (*SD*)	*M* (*SD*)	*M* (*SD*)	*M* (*SD*)
No weekly non-parental care arrangement (*n* = 2,036)	92.87 (25.08)	35.15 (9.05)	26.85 (9.79)	30.86 (9.78)
Weekly non-parental care arrangement (*n* = 1,806)	91.58 (26.25)	35.07 (9.51)	26.06 (10.29)	30.44 (9.84)
F statistics	2.432	0.070	5.978*	1.759
Partial η^2^	–	–	0.002	–

*PSI-SF, Parenting Stress Index – Short Form; PD, Parental Distress subscale; P-CDI, Parent-Child Dysfunctional Interaction subscale; DC, Difficulty Child subscale.*

*p < 0.05.

## Discussion

Over the past few decades, participation in non-parental child care or early childhood programs has become commonplace for many Chinese children. However, there has not been much focus on families with infants and toddlers. Especially since infant-toddler child care service has become a key factor restricting family fertility willingness, it has been considered an important supporting measure in the implementation of China’s three-child policy. Given the emphasis in recent years on infant-toddler child care service under China’s population strategy, it is critical to gain a better understanding of where children are spending their time before preschool entry. With a focus on infants and toddlers, this study explored the patterns of non-parental care arrangements participation, parental preferences and constraints in finding nurseries, as well as the association between the utilization of non-parental child care and parenting stress. This study, based on a large survey of non-parental child care arrangements in mainland China, fills in the gaps in relevant research and enriches the research on non-parental child care for children under age 3. In addition, this study provides new evidence for the relationship between non-parental child care and parenting stress after the adjustment of China’s fertility policy and offers a direction for follow-up research on how to provide targeted fertility and family support for Chinese families with very young children.

### Prevalence of Non-parental Child Care Arrangements

Our findings show that approximately 47.0% of children from birth through age 3 received weekly non-parental child care, and non-parental care arrangement was predominantly informal home-based care provided by relatives such as grandparents in mainland China, which is consistent with previous evidence that Chinese families rely heavily on grandparents to care for their children (Chen et al., 2011). With the introduction of the two-child policy and the new three-child policy, many Chinese families are facing an even more severe issue of “no one to take care of their children,” especially at present, women’s participation in the labor force market is increasing, which makes this problem more urgent. Faced with a huge child care dilemma, many families request grandparents to take care of their children (Hong and Zhu, 2019). In accordance with patrilineal customs, paternal grandparents in mainland China are usually more likely than maternal grandparents to care for their grandchildren (Chen et al., 2000). Furthermore, research suggests numerous features of families can predict the parental need for non-parental care, as well as their resources to access child care services (e.g., Meyers and Jordan, 2006). Findings from this study support these claims. In this study, the labor force status of parents, household income, parental education, and mother’s age were significant predictors of very young children’s participation in non-parental child care arrangements. Parents with higher education, at a younger age, or from higher income and dual-earner families were at higher rates in the utilization of non-parental child care arrangements. Among them, younger parents were more able to accept non-parental child care and pursue personal freedom and liberation, whereas many older parents worried that their children were too little to enter the nursery. Moreover, parents with higher education, or from higher income families, generally had higher social status; meanwhile, they normally perceived greater levels of stress and more conflicts between work and parenting. Given gender inequality in household labor, women typically bear a disproportionate burden in child care responsibilities at home. Especially for highly educated working mothers, pregnancy, childbirth, and child-rearing might add a negative consequence to their career development. As a result, they were more eager to use non-parental child care arrangements.

### Parental Priority in and Demand for Child Care Services

Non-parental child care arrangements are typically used for two main reasons: (1) parents’ participation in the workforce and (2) children’s growth and development (Kim and Fram, 2009). With respect to the key reason for wanting a care program, in contrast with studies indicating parents in Western countries typically need child care due to their employment, study, or personal responsibilities (Corcoran and Steinley, 2019), Chinese parents placed great emphasis on early learning of their children. This might be due to Chinese parents usually holding high expectations for their children’s educational achievement (Settles et al., 2008). Non-parental child care is more likely to be considered by Chinese parents as a strategy to promote their children’s growth and development, rather than as only support for parental employment. Another possible explanation might be related to the age group of children involved in this study; more than half of them were 3- to 4-year-old; at this age, children are expected to acquire preschool readiness skills.

The country has not invested successfully in infant-toddler child care for decades, resulting in a shortage of affordable and high-quality options (Qi and Melhuish, 2017). Currently, most Chinese families live in child care deserts, where there is an insufficient supply of nurseries. As the infant-toddler child care service system in mainland China is still in its infancy, it is not surprising that nearly 70% of parents reported difficulty finding nurseries; parents were facing numerous barriers to accessing nurseries, and the quality of care in nurseries was rated as the top concern for parents in finding and choosing nurseries. In mainland China, public early childhood programs (e.g., preschools) typically represent affordable and high-quality child care services. Therefore, the majority of Chinese parents preferred publicly funded nurseries and expected preschool slots to accommodate infants and toddlers. Policies must address the problems of supply, affordability, and quality of infant-toddler child care services in mainland China.

Findings from this present study indicate that nurseries were needed most for children between 31 and 36 months as reported by parents. In mainland China, the first day of the school year is typically September 1 across the country, which is considered the nationwide school cutoff date (Zhang and Xie, 2018). Most Chinese preschools only admit children who have already reached their third birthday on or before September 1 that year, while those born after September 1 of that year should wait until the next school year. Therefore, Chinese parents of older toddlers in that age group have a particularly high demand for child care services, especially when their children are not age eligible for preschool and have to wait another year due to the age requirement for preschool enrollment.

### Parenting Stress in Families of Very Young Children

The utilization of non-parental child care arrangements has the potential to ameliorate the psychological tensions of parenthood (Craig and Churchill, 2018). However, this study found the utilization of non-parental child care arrangements was not significantly linked to parenting stress in terms of parental distress and their views about a child’s difficult behaviors. Results also indicated that parents of children who participated in non-parental child care arrangements were slightly less stressed in interacting with their children than parents who did not use non-parental child care. On the one hand, these findings support prior research, indicating that young children’s participation in non-parental child care was not linked to a reduction in parenting stress due to the associated demands and concerns on child care (e.g., Magnuson and Waldfogel, 2005; Craig and Churchill, 2018). On the other hand, these results should be interpreted with caution, given the intensity of non-parental child care (e.g., the average length of child care per week) was not assessed in this study, and the types of non-parental child care arrangements were not distinguished. It might be possible that parents will experience less parenting stress with an increased intensity of grandparental child care (Wheelock and Jones, 2002). Regardless of whether or not children participate in non-parental child care arrangements, considerable efforts should be made to improve parenting capabilities and family resilience.

### Limitations and Implications

Currently, the Chinese government has put great efforts into boosting infant-toddler child care services. This study provides the most recent snapshot of non-parental child care arrangements for children from birth through 3 years of age under China’s new family planning policy. However, several limitations should be mentioned. First, the sample for this study was recruited from 10 provinces in mainland China and was predominantly parents of older toddlers, who might not be representative of the population of Chinese parents across mainland China. Children’s non-parental care arrangements and parental demand for child care might vary by children’s ages. Second, this study did not differentiate the types of non-parental child care arrangements, as well as the intensity of non-parental child care. Third, this study did not examine child outcomes related to children’s participation in non-parental child care arrangements. Future studies are needed to investigate the connections between child outcomes and non-parental child care experience in the Chinese sociocultural context.

Nevertheless, our findings contribute to a better understanding of the unitization of non-parental child care arrangements in mainland China and have several implications for government policymaking in Chinese societies. In the era of China’s three-child policy, the provision of child care services should promote child development and well-being, as well as support parents’ labor force participation and family fertility. As the Chinese government is establishing its infant-toddler child care system, infant-toddler child care policies should take family backgrounds and needs into consideration, adapting to the evolving needs of very young children and their families. First, given many Chinese families rely on grandparental child care, family support should be provided to both parents and grandparents through training, counseling, and other activities, with a focus on improving the parent-grandparent co-parenting relationship. Second, understanding families’ preferred characteristics of care are of paramount importance with respect to policy decisions. The types of non-parental child care arrangements should be diversified based on families’ preferences and needs. Third, this study also showed many Chinese families with very young children were experiencing difficulty in finding a nursery under the current system. The growing demand for nurseries for children under age 3 needs to be met with an investment to expand the supply of services. The country must give priority to the needs of working families and promote access to affordable and high-quality child care services for infants and toddlers before they enter preschool.

## Data Availability Statement

The raw data supporting the conclusions of this article will be made available by the authors, without undue reservation.

## Ethics Statement

The studies involving human participants were reviewed and approved by Beijing Normal University. Written informed consent for participation was not required for this study in accordance with the National Legislation and the Institutional Requirements.

## Author Contributions

XH, WZ, and LL did conceptualization and design of the research. All authors contributed to the data analyses and interpretation, and involved in the manuscript writing.

## Conflict of Interest

The authors declare that the research was conducted in the absence of any commercial or financial relationships that could be construed as a potential conflict of interest.

## Publisher’s Note

All claims expressed in this article are solely those of the authors and do not necessarily represent those of their affiliated organizations, or those of the publisher, the editors and the reviewers. Any product that may be evaluated in this article, or claim that may be made by its manufacturer, is not guaranteed or endorsed by the publisher.

## Footnotes

1 In the questionnaire, parents could choose more than one main reason for wanting a nursery.
